# Fixation stability and stress redistribution following metal block use in opening-wedge high tibial osteotomy: a finite element analysis

**DOI:** 10.3389/fbioe.2025.1703140

**Published:** 2025-12-17

**Authors:** Kyung-Wook Nha, Hyungsuh Kim, Jae-Gwang Song, Hyongtaek Hong, Kyoung-Tak Kang, Hyung Jun Park

**Affiliations:** 1 Department of Orthopedic Surgery, Ilsanpaik Hospital, Inje University College of Medicine, Goyang, Gyeonggi-do, Republic of Korea; 2 Department of Orthopedic Surgery, Suncheon Joongang Hospital, Suncheon, Jeollanam-do, Republic of Korea; 3 Skyve R&D Lab, Seoul, Republic of Korea; 4 Department of Mechanical Engineering, Yonsei University, Seoul, Republic of Korea; 5 Department of Orthopedic Surgery, Korea University Ansan Hospital, Korea University College of Medicine, Ansan, Gyeonggi-do, Republic of Korea

**Keywords:** opening wedge high tibial osteotomy (OWHTO), metal block augmentation, lateral hinge fracture, biomechanical stability, finite element analysis

## Abstract

**Introduction:**

Medial opening wedge high tibial osteotomy (OWHTO) is a widely performed procedure for correcting varus malalignment and alleviating medial compartment osteoarthritis. Metal block augmentation has been proposed to enhance construct stability by reducing micromotion and stress at the osteotomy site. However, its biomechanical effects under lateral hinge fracture (LHF) and across different osteotomy techniques (uniplanar vs. biplanar osteotomy) remain poorly understood.

**Methods:**

A finite element model of the proximal tibia was constructed using the computed tomography data of a 62-year-old woman. Simulations were conducted under uniplanar and biplanar osteotomy configurations, with and without a 12 mm metal block augmentation. The LHF was modeled for three Takeuchi fracture types, in addition to the intact condition. Each model was evaluated under axial loading to quantify micromotion, peak stress at the D-hole, mean stress at the lateral hinge, and stress distribution in the locking plate and the proximal tibia.

**Results:**

Metal block augmentation significantly improved the fixation stability across all OWHTO configurations. In the uniplanar models, the micromotion was reduced by over 90% in both the non-fracture and Type I LHF conditions, whereas the reduction ranged from 84% to 91% in the biplanar models. The peak stress around the D-hole decreased by 14%–21% in constructs with a metal block compared to those without. However, the mean plate stress increased substantially by 87% in the uniplanar model and 237% in the biplanar model. In contrast, the proximal tibial bone stress consistently decreased by 21%–28%.

**Conclusion:**

Metal block augmentation improved the biomechanical stability in OWHTO constructs, with greater grains in uniplanar osteotomies and LHF models. This enhancement was accompanied by altered stress distribution, characterized by increased stress on the plate and reduced stress in the proximal tibia, suggesting a potential stress-shielding effect. By quantifying these effects under various conditions, this study provides biomechanical evidence for the selective application of metal block augmentation in clinical practice.

## 1 Introduction

Medial opening wedge high tibial osteotomy (OWHTO) is a widely adopted procedure for correcting varus malalignment and alleviating medial compartment osteoarthritis (Jin et al., 2021; Palmer et al., 2024). According to the International Society of Arthroscopy, Knee Surgery and Orthopedic Sports Medicine (ISAKOS), ideal candidates are high-demand individuals aged 40–60 years who maintain a moderately active lifestyle (Rand and Neyret, 2005). Reflecting its increasing clinical adoption, the number of OWHTO has risen nearly 6-fold over the past decade in Korea (Lee et al., 2021). In this economically active population, early return to function is essential not only for individual postoperative recovery but also for minimizing productivity loss at a societal level (Ademi et al., 2021). Early rehabilitation after OWHTO has been associated with a lower incidence of complications such as thrombophlebitis and more rapid recovery of knee function within a shorter postoperative period (Lee et al., 2017; Gupta et al., 2024). These clinical demands have driven the evolution of surgical techniques and implant designs aimed at enabling full weight-bearing at an earlier stage (Agneskirchner et al., 2006; Smith et al., 2013). Earlier approaches to OWHTO, including monoplanar osteotomy and non-locking fixation constructs, were limited by insufficient mechanical stability, leading to delayed rehabilitation and increased risk of correction loss or implant failure (Lobenhoffer and Agneskirchner, 2003; Stoffel et al., 2004; Diffo Kaze et al., 2015; Han et al., 2017). In response, biplanar osteotomy has been introduced to maximize the surface contact and enhance initial mechanical stability (Lobenhoffer and Agneskirchner, 2003). Additionally, locking plate systems were developed to overcome the shortcomings of conventional non-locking constructs, allowing for accelerated postoperative rehabilitation as early as 2 weeks after surgery (Stoffel et al., 2004; Diffo Kaze et al., 2015; Han et al., 2017; Lee et al., 2017; Schroter et al., 2017; van Haeringen et al., 2023). To further enhance the stability required for early weight-bearing, recent innovations have focused on two features (Zhao et al., 2022; Park et al., 2024). First, the integration of a metal block into the osteotomy gap has been proposed to reinforce fixation by reducing micromotion and directly transmitting compressive forces across the opening gap (Han et al., 2014). Second, new locking plate designs incorporate a proximal four-screw configuration that reinforces the structurally weak area surrounding the D-hole, which is a known point of mechanical vulnerability (Agneskirchner et al., 2006; Park et al., 2024).

Lateral hinge fracture (LHF) is a recognized complication of OWHTO, as the lateral cortex serves as a mechanical fulcrum essential for stability (Choi et al., 2021; van Haeringen et al., 2023; Hung et al., 2024). The disruption of this structure compromises the load-sharing mechanism, resulting in increased opening of the osteotomy gap and excessive stress concentration on the plate and screws (Kang et al., 2020; Palmer et al., 2024). Consequently, maintaining hinge integrity or reinforcing stability becomes critical, particularly in cases involving large correction angles or an increased posterior tibial slope, both of which increase the LHF risk (Han et al., 2013; Kang et al., 2020; Winkler et al., 2021; Song et al., 2022). A recent finite element study indicated that metal block augmentation reduces the stress on the locking plate and lateral hinge (Jang et al., 2018). However, its stabilizing effect in the presence of lateral hinge fracture has not been sufficiently studied. Moreover, in clinical practice, the choice between uniplanar and biplanar osteotomies is often determined by the correction angle or the need for slope adjustment (Pornrattanamaneewong et al., 2012; Luo et al., 2013; Yoo et al., 2016). However, whether metal block augmentation offers consistent biomechanical benefits across these techniques remains unclear.

Therefore, this study aimed to evaluate the biomechanical effects of metal block augmentation in OWHTO and determine whether its stabilizing role differs according to the presence of LHF and the type of osteotomy. We hypothesized that metal block augmentation would enhance the biomechanical stability of the osteotomy construct, regardless of the presence of an LHF, with a more pronounced stabilizing effect observed in uniplanar osteotomies compared to biplanar configurations. Furthermore, we postulated that the application of a metal block would alter the load transmission patterns, leading to stress redistribution in the plate and surrounding bone.

## 2 Materials and methods

### 2.1 Development of a finite element model of the proximal tibia

Cross-sectional images of the lower limbs of a 62-year-old Asian woman were acquired using a 64-channel computed tomography (CT) scanner (Somatom Sensation 64; Siemens Healthcare, Erlangen, Germany) with a slice thickness of 0.1 mm and a slice interval of 0.1 mm. Based on these images, a three-dimensional model of the proximal tibia was reconstructed in Mimics (version 21.0; Materialise Inc., Belgium), excluding the fibula from the final finite element (FE) model. The surface geometry was refined and converted into solid structures using Unigraphics NX (version 7.0; Siemens PLM Software, Torrance, CA, United States), and the FE mesh was developed using HyperMesh version 8.0 (Altair Engineering, Troy, MI) (Park et al., 2024). The study protocol was approved by our institutional review board.

### 2.2 Simulation of opening wedge high tibial osteotomy with plate and screw fixation

**FIGURE 1:**
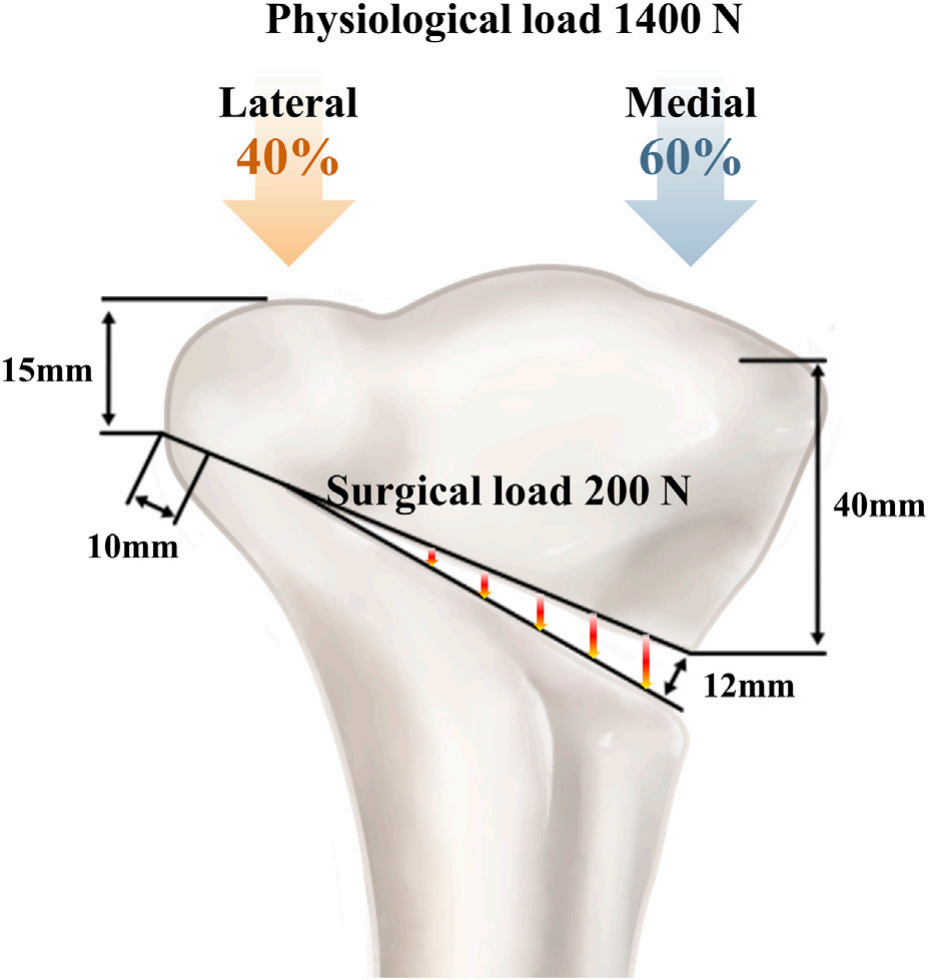
Finite element models of opening wedge high tibial osteotomy with and without metal block augmentation. A 12 mm medial opening wedge was created starting 40 mm distal to the knee joint line and extended to a point 15 mm distal to the lateral tibial plateau, preserving a 10 mm lateral cortical hinge.

**FIGURE 2:**
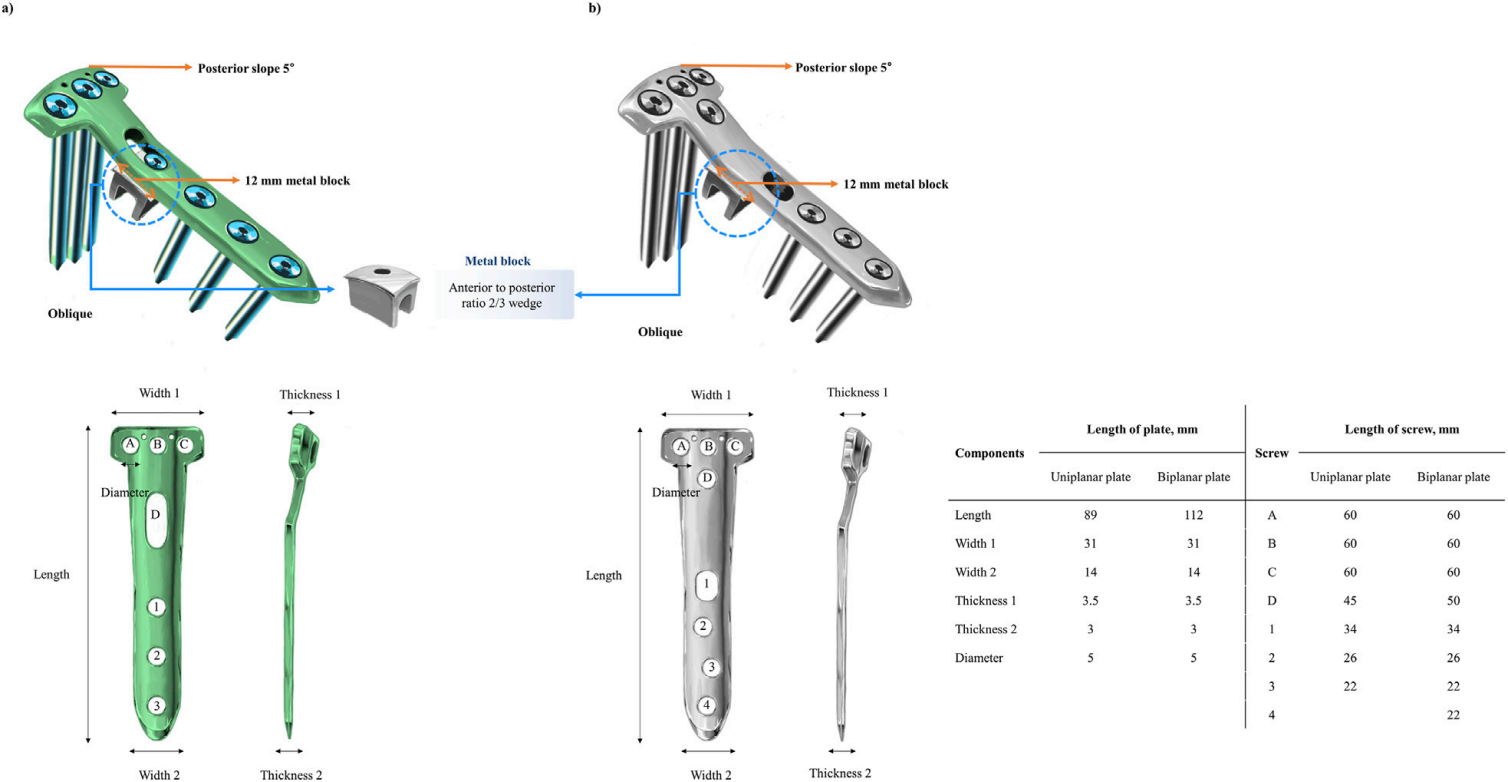
Metal block geometry, configurations, and dimensions of the locking plate and screws. Uniplanar **(a)** and biplanar **(b)** locking plate constructs with a 12 mm metal block seated in the osteotomy gap, contoured with a 2:3 anterior to posterior height ratio to preserve the native 5° posterior tibial slope. Configurations and dimensions of the locking plates and screws are shown.

To simulate OWHTO, the distal segment of the proximal tibia was rotated laterally in the coronal plane to achieve valgus alignment (Fujisawa et al., 1979). This alignment was achieved by creating a 12 mm opening wedge at the posteromedial tibial corner. The osteotomy started 40 mm distal to the knee joint line and extended to a point 15 mm distal to the lateral tibial plateau, preserving a 10 mm lateral cortical hinge (Yoon et al., 2023) (Figure 1). Surgical simulation was performed under clinical guidance to ensure anatomical accuracy (Noyes et al., 2000; Hankemeier et al., 2006). Postoperative FE models were generated for uniplanar and biplanar OWHTO configurations. An OhtoFix plate (Ohtomecal; Goyang-si, Gyeonggi-do, Republic of Korea) was implanted for fixation. Two fixation conditions were modeled: one using a standard locking plate and screws without a metal block, and the other incorporating a 12 mm height metal block precisely contoured to fit the internal geometry of the plate, with an anterior-to-posterior height ratio of 2:3 to maintain the native posterior tibial slope (Chae et al., 2008; Han et al., 2014) (Figure 2). These models were created using SolidWorks (version 2023 SP5.0; Dassault Systèmes, Velizy-Villacoublay, France). The detailed configuration of the locking plate and the lengths of all screws used in the fixation are specified in Figure 2 to ensure reproducibility and design clarity.

**FIGURE 3:**
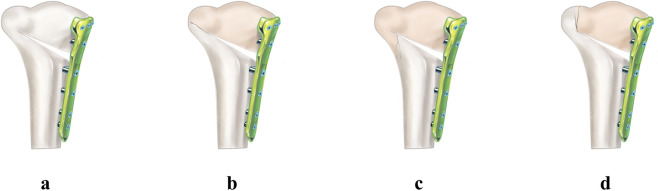
Classification of lateral hinge fracture according to the Takeuchi classification. **(a)** Intact lateral cortex. **(b)** Type I fracture. **(c)** Type II fracture. **(d)** Type III fracture.

**FIGURE 4:**
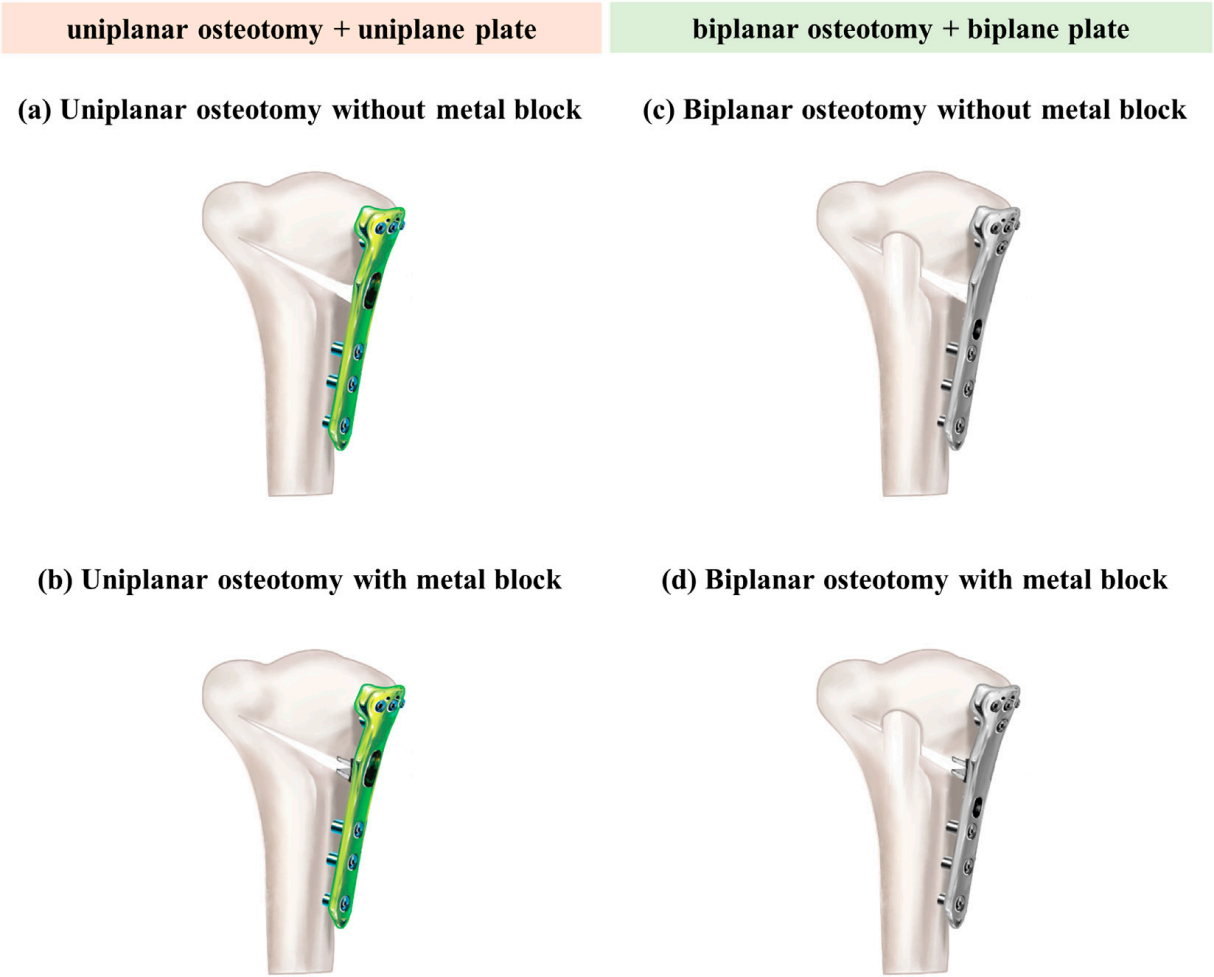
Finite element models of opening wedge high tibial osteotomy according to osteotomy type and fixation method. **(a)** Uniplanar osteotomy without a metal block. **(b)** Uniplanar osteotomy with a metal block. **(c)** Biplanar osteotomy without a metal block. **(d)** biplanar osteotomy with a metal block.

To evaluate the biomechanical effects of LHF, three fracture types were modeled according to the Takeuchi classification—Type I, Type II, and Type III—and an intact lateral hinge condition (Takeuchi et al., 2012) (Figure 3). Type I is characterized by a fracture line extending from the osteotomy site to the lateral cortex of the proximal tibia, resulting in minimal instability. Type II involves a fracture that extends distally. Type III fractures extend into the lateral tibia plateau, resulting in significant instability (Takeuchi et al., 2012). These fracture configurations were applied to four osteotomy-fixation constructs: (1) uniplanar osteotomy without a metal block, (2) uniplanar osteotomy with a metal block, (3) biplanar osteotomy without a metal block, and (4) biplanar osteotomy with a metal block. A total of 16 finite element models were generated and analyzed (Figure 4).

### 2.3 Material property

The material properties for cortical bone, cancellous bone, plates, and screws were based on previous studies (Morgan et al., 2003; Luo et al., 2013; Luo et al., 2015). The cortical bone was considered as a linear elastic, isotropic, and homogeneous material with a Young’s modulus of 17,000 MPa and Poisson’s ratio of 0.33 (Luo et al., 2013; Luo et al., 2015). The cancellous bone has a modulus of 910 MPa and Poisson’s ratio of 0.2 (Luo et al., 2015). The plates and screws were made of Ti-6A-l4V ELI with a modulus of 110 GPa and a Poisson’s ratio of 0.3 (Morgan et al., 2003) (Table 1).

**TABLE 1:** Material properties of model components.

Components	Young’s modulus, GPa	Poisson’s ratio, ν
Cortical bone	17	0.33
Cancellous bone	0.91	0.20
Ti-6A-l4V ELI	110	0.30

Young’s modulus, Poisson’s ratio; all materials modeled as linear elastic, homogeneous, and isotropic.

### 2.4 Loading and boundary conditions

Two loading conditions were applied to the OWHTO models to replicate surgical intervention and physiological weight-bearing (Luo et al., 2013; Koh et al., 2019). A compression load of 200 N was used to simulate the forces generated by soft-tissue tension, including the medial collateral ligament, the patellar tendon, and the remaining intact lateral cortex (Blecha et al., 2005). This load was distributed uniformly across the osteotomy gap to represent intraoperative stabilization. In addition, a physiological axial load of 1,400 N was applied to the tibial plateau to mimic joint loading during the stance phase. The axial load was distributed at a medial-to-lateral ratio of 60:40, based on previous studies that reported 62%–75% of the load on the medial compartment (Figure 1) (Koh et al., 2019). A fully bonded (tie) contact condition was assumed between all interfaces, including bone-bone and bone-implant regions. The distal end of the tibia was assumed to be fully fixed in all tests (Gray et al., 2008; Cho et al., 2022). All simulations were conducted under static, single-cycle loading conditions rather than dynamic cyclic loading to reflect the peak mechanical environment encountered during early postoperative weight-bearing.

### 2.5 Outcome measures

Four primary biomechanical parameters were evaluated to assess the fixation stability and stress distribution in the OWHTO constructs. First, micromotion at the osteotomy gap was measured to quantify the mechanical instability and potential for displacement at the opening site. Second, the peak von Mises stress was recorded around the D-hole of the locking plate, a region known for its mechanical vulnerability, to assess the localized stress concentration (4, 13). Third, the mean von Mises stress was calculated in the lateral hinge region of the proximal tibia to evaluate the stress transfer across the osteotomy fulcrum. Finally, the mean von Mises stress was determined across the entire locking plate and the proximal tibia to investigate stress redistribution and potential stress-shielding effects. All outcomes were extracted from the static, single-cycle loading simulations described above. No time-dependent material behavior or cyclic loading protocol was implemented. To contextualize the stress values, the yield strength of the cortical bone was referenced as 177.2 MPa under axial compression (30). All the simulations were performed using ABAQUS (version 6.14; Dassault Systèmes, France).

### 2.6 Mesh convergence

Mesh convergence was confirmed when the calculated displacement values in the trabecular bone deviated by less than 5% from those obtained using finer mesh resolutions (Kwon et al., 2017). To ensure computational precision, a mesh size of 1.0 mm was applied to the regions surrounding the osteotomy plate and screw holes. In contrast, a slightly coarser mesh size of 1.2 mm was used for all the other areas. All components were discretized with quadratic tetrahedral elements (C3D10). In the final models, the cortical bone, cancellous bone, plate, and screws consisted of approximately 232 k, 555 k, 30 k, and 46 k elements, respectively, with corresponding node counts of 346 k, 829 k, 46 k, and 75 k.

### 2.7 Intact model validation

The FE model was validated against data from a previous study, demonstrating the experimental validation of a finite element model of a human cadaveric tibia (Gray et al., 2008). Validation was performed under torsional loading conditions, with a focus on the minimum and maximum principal strains. The highest minimum principal strains observed in the tibia bone and the reference model were −542 and −569 microstrain, respectively. The maximum numbers of principal strains were 403 and 426 microstrains, respectively. The differences in the strain values were less than 10%. Consequently, the FE model used in this study was validated by comparing the maximum and minimum principal strains with those from the reference data.

## 3 Results

### 3.1 Fixation stability enhancement by metal block

**FIGURE 5:**
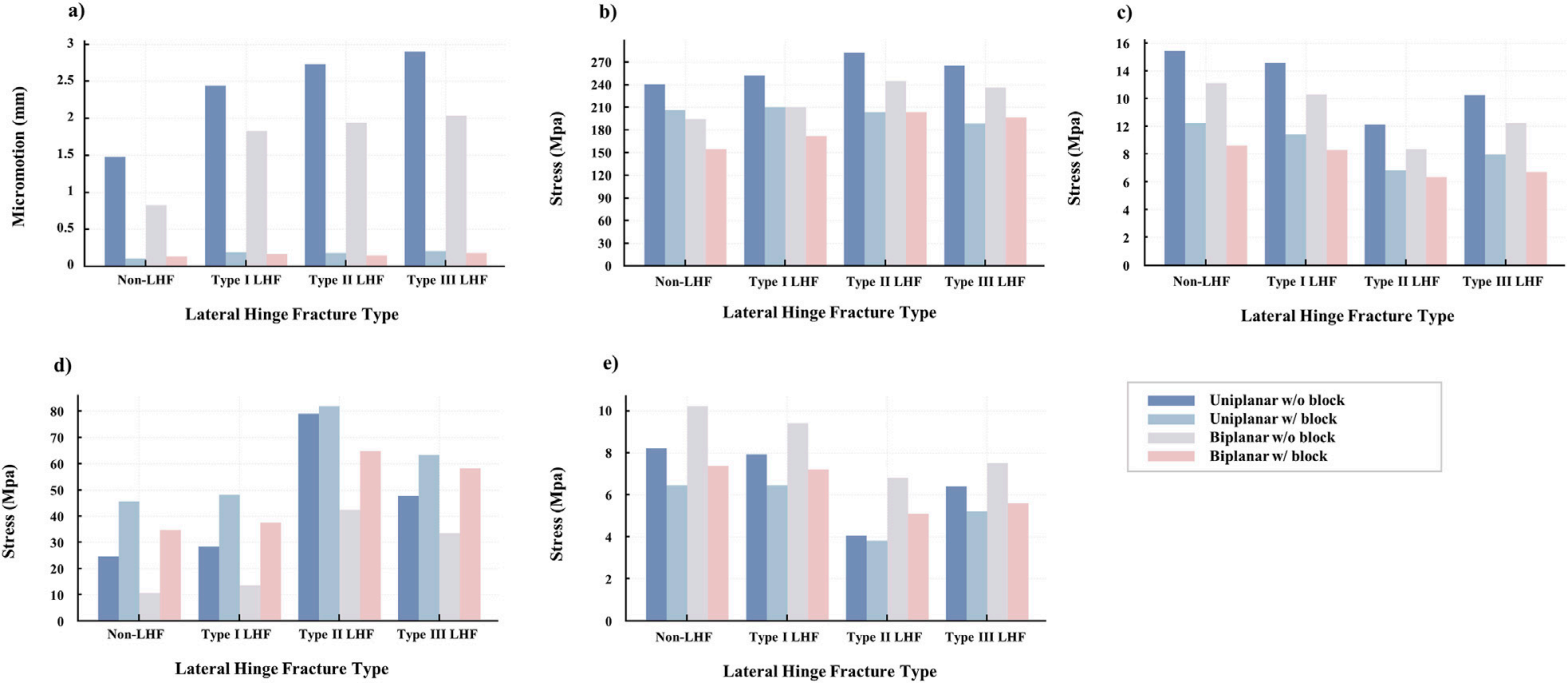
Comparison of biomechanical parameters across opening wedge high tibial osteotomy configurations according to the osteotomy type (uniplanar and biplanar), metal block use, and lateral hinge fracture status. **(a)** Micromotion (mm). **(b)** Peak von Mises stress at the D-hole of the plate (MPa). **(c)** Mean von Mises stress at the lateral hinge (MPa). **(d)** Mean von Mises stress in the locking plate (MPa). **(e)** Mean von Mises stress in the proximal tibial bone (MPa).

Incorporating a metal block into OWHTO constructs consistently enhanced the biomechanical stability across all evaluated configurations. This effect was more pronounced in uniplanar osteotomies, which exhibited lower baseline stability. Micromotion at the osteotomy gap was significantly reduced with metal block augmentation, regardless of the osteotomy type or presence of a lateral hinge fracture. In the non-hinge fractured model, the micromotion decreased by 93.2% in the uniplanar model (from 1.47 to 0.10 mm) and by 84.1% in the biplanar model (from 0.82 to 0.13 mm). In Type I LHF, micromotion was reduced by 92.6% in the uniplanar model (from 2.44 to 0.18 mm) and by 90.7% in the biplanar model (from 1.83 to 0.17 mm; Table 2; Figure 5a).

**TABLE 2:** Micromotion, peak von Mises stress at the D-hole, and mean stress at the lateral hinge according to osteotomy type, metal block use, and lateral hinge fracture status.

Osteotomy type	Metal block	Micromotion, mm				Peak von Mises stress at D-hole, MPa				Mean stress at lateral hinge, MPa			
		Non-LHF	Type I LHF	Type II LHF	Type III LHF	Non-LHF	Type I LHF	Type II LHF	Type III LHF	Non-LHF	Type I LHF	Type II LHF	Type III LHF
Uniplanar	No	1.47	2.44	2.73	2.89	241	252	283	264	15.4	14.5	10.1	12.2
Uniplanar	Yes	0.10	0.18	0.17	0.20	206	210	204	189	10.2	9.4	6.8	7.9
Biplanar	No	0.82	1.83	1.94	2.03	195	211	245	236	13.1	12.2	8.3	10.2
Biplanar	Yes	0.13	0.17	0.15	0.17	154	172	203	197	8.6	8.3	6.3	6.7

Abbreviation: LHF, lateral hinge fracture.

**FIGURE 6:**
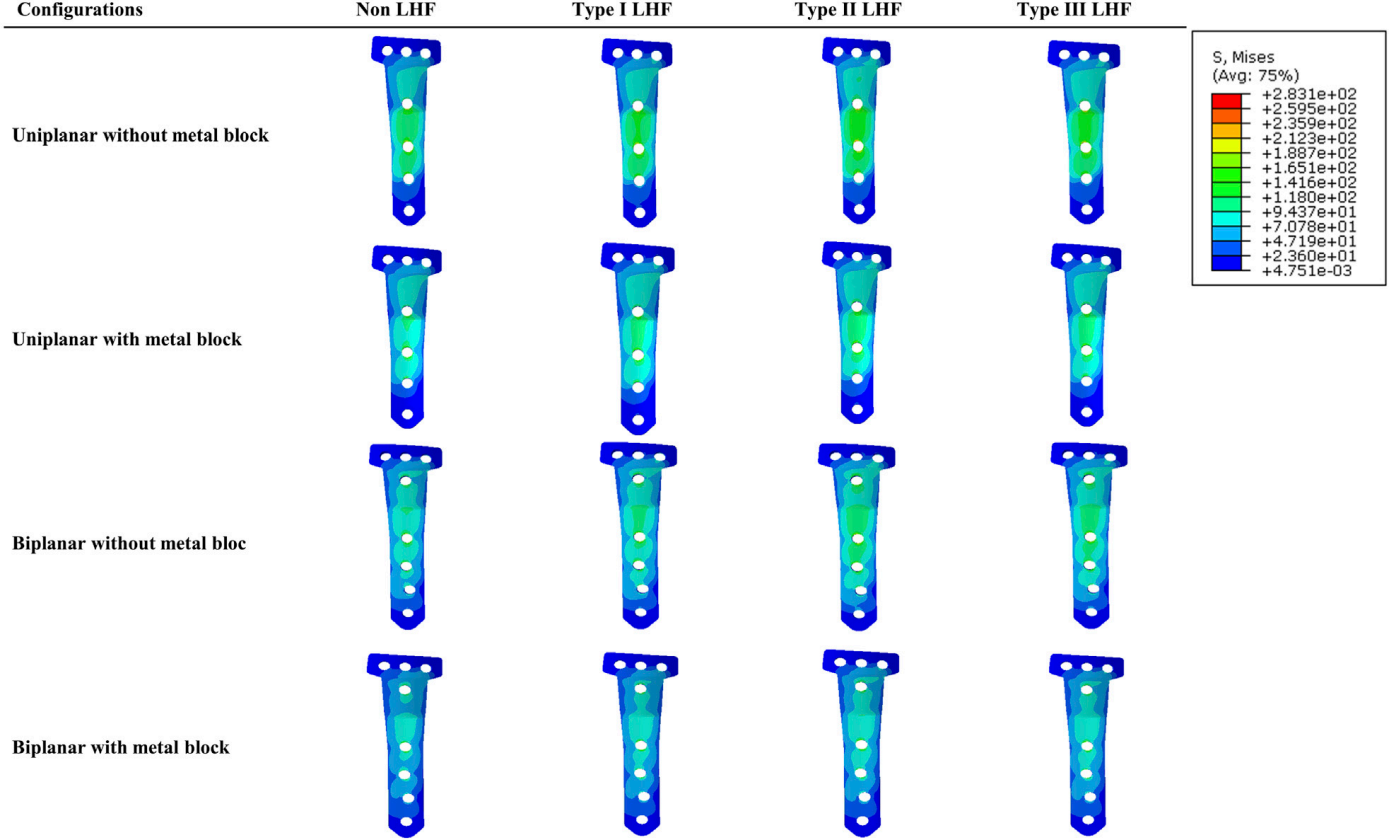
Peak Von Mises stress distribution in the locking plate by osteotomy type and the presence of a metal block.

Additionally, the peak von Mises stress at the D-hole decreased after the metal block augmentation (Figure 6). In the non-hinge fractured model, the stress decreased by 14.5% in the uniplanar model (from 241 to 206 MPa) and by 21.0% in the biplanar model (from 195 to 154 MPa, Figure 5b). Similarly, the average stress transferred to the lateral hinge decreased when a metal block was used. In the Type I LHF model, the hinge stress declined by 35.2% in the uniplanar model (from 14.5 to 9.4 MPa) and by 32.0% in the biplanar model (from 12.2 to 8.3 MPa; Table 2; Figure 5c).

### 3.2 Stress redistribution and stress shielding effects

Metal block augmentation redistributes mechanical stress, with increased stress on the locking plate and decreased stress in the proximal tibia. In the non-hinge fractured model, the mean plate stress increased by 87.2% in the uniplanar model (from 24.3 to 45.5 MPa) and by 236.9% in the biplanar model (from 10.3 to 34.7 MPa, Table 3; Figure 5d). A similar trend was observed in the models with LHF, with the most pronounced increase observed in the Type II LHF model. Conversely, the average stress within the proximal tibia was consistently reduced following metal block use. In the uniplanar non-fracture model, the stress decreased by 20.7% (from 8.2 to 6.5 MPa) and by 27.5% in the biplanar model (from 10.2 to 7.4 MPa; Table 3; Figure 5e).

**TABLE 3:** Mean von Mises stress in the locking plate and proximal tibial bone according to the osteotomy type, metal block use, and lateral hinge fracture status.

Osteotomy type	Metal block	Mean stress in locking plate, MPa				Mean stress in proximal tibia, MPa			
		Non-LHF	Type I LHF	Type II LHF	Type III LHF	Non-LHF	Type I LHF	Type II LHF	Type III LHF
Uniplanar	No	24.3	28.2	79.0	47.8	8.2	7.9	4.0	6.4
Uniplanar	Yes	45.5	47.9	81.7	63.4	6.5	6.4	3.8	5.2
Biplanar	No	10.3	13.4	42.5	33.4	10.2	9.4	6.8	7.5
Biplanar	Yes	34.7	37.3	64.7	58.2	7.4	7.2	5.1	5.6

Abbreviation: LHF, lateral hinge fracture.

## 4 Discussion

OWHTO is a widely used procedure for treating varus knee osteoarthritis, particularly in young and active patients (Jin et al., 2021; Palmer et al., 2024). Achieving a rigid and stable fixation at the osteotomy site is crucial to support early weight-bearing (Agneskirchner et al., 2006; Smith et al., 2013). Various techniques, including biplanar osteotomy, locking plates, and metal block augmentation, have been introduced to improve construct stability (Lobenhoffer and Agneskirchner, 2003; Stoffel et al., 2004; Diffo Kaze et al., 2015; Han et al., 2017). Although metal blocks have been shown to reduce micromotion and stress around the medial plate and lateral hinge, most previous studies were limited to models without LHF and did not comprehensively assess the different types of osteotomy (Han et al., 2014; Jang et al., 2018). Moreover, the combined influence of osteotomy configuration (uniplanar vs. biplanar) and LHF on fixation stability has not been adequately studied. The principal finding of our study was that metal block augmentation enhanced the biomechanical stability across all configurations, particularly in uniplanar osteotomies and in the presence of LHF. However, these biomechanical advantages may be accompanied by altered stress distributions within the locking plate and the proximal tibia, suggesting a potential trade-off between mechanical reinforcement and biological load-sharing.

Our results confirmed the hypothesis that metal block augmentation improves the biomechanical stability of OWHTO constructs regardless of the osteotomy type or the presence of a lateral hinge fracture. Uniplanar osteotomies are inherently less stable than biplanar techniques and more vulnerable to lateral hinge instability (Lobenhoffer and Agneskirchner, 2003; Palmer et al., 2024). Additionally, a lateral hinge fracture can result in translational and rotational displacement between the two fragments, thereby compromising or delaying bone healing (Palmer et al., 2024). Although biplanar osteotomy is generally favored for its superior structural integrity, uniplanar techniques remain a necessary option in specific clinical cases, such as those involving patients with preexisting patella baja or when concurrent procedures (e.g., double-level osteotomy) are planned, where a shorter operative time is desirable (Zheng et al., 2022). In these situations, metal block augmentation offers a viable strategy that enables the performance of uniplanar osteotomy while reducing the risk of hinge fractures and improving fixation strength, as supported by previous biomechanical studies (Han et al., 2014; Jang et al., 2018; Nha et al., 2020; Hung et al., 2024). An FE analysis reported that reduced stress transmission to the lateral hinge with a metal block (Jang et al., 2018). Another clinical study, although employing a structural allograft rather than a metal block, demonstrated a successful bone union regardless of the hinge fracture status, supporting the concept that structural reinforcement at the osteotomy site can compensate for mechanical deficiencies (Hung et al., 2024). As shown in Table 2, our results also demonstrated that lateral hinge fracture substantially increased the micromotion, particularly in uniplanar constructs. However, metal block augmentation consistently reduced the micromotion across all subtypes. After augmentation, absolute micromotion values were uniformly low, indicating that the relative benefit was most pronounced in the uniplanar models, because of their lower baseline stability. This phenomenon is mechanically plausible: by occupying the osteotomy gap, the metal block restores direct medial-lateral contact, creates a load-transferring interface across the osteotomy plane, and increases construct stiffness, thereby reducing interfragmentary motion under axial loading and attenuating rotational instability. Lateral hinge fracture also redistributed peri-implant stress, elevating stress around the D-hole while reducing stress at the lateral hinge. The insertion of the metal blocks attenuated these stress concentrations. In the uniplanar Type I fracture model, the D-hole stress with metal block augmentation approximated the levels observed in the non-metal block-augmented biplanar models. Collectively, these findings suggest that metal block augmentation improves load transfer and suppresses rotational instability, producing a disproportionate benefit in the uniplanar construct. From a practical standpoint, when simplified or time-efficient surgical strategies are required, an uniplanar approach combined with metal block augmentation could be a viable option. Nonetheless, because reinforcement may alter local load sharing—such as stress shielding or increased plate stress—careful plate-block design and longer-term clinical validation are warranted.

Our results confirmed the hypothesis that metal block augmentation increases the stress on the locking plate while concurrently reducing the stress on the surrounding bone, thereby inducing a stress-shielding effect. When performing OWHTO, enhancing fixation strength is a significant factor in facilitating early weight-bearing and postoperative rehabilitation (Agneskirchner et al., 2006; Smith et al., 2013). However, increased construct stiffness may unintentionally reduce physiological stress transmission to the surrounding bone, a phenomenon known as stress shielding (Roderer et al., 2014; Xu et al., 2025). Previous studies highlighted the biological consequences of excessive implant rigidity (Laftman et al., 1989; Roderer et al., 2014; Park et al., 2021; Xu et al., 2025). One study reported that the application of locking plates significantly reduced new bone formation at the osteotomy site (Roderer et al., 2014). Similarly, another study demonstrated enhanced bone healing around the osteotomy site after plate removal, suggesting that stress shielding is reversible once the mechanical obstruction is eliminated (Park et al., 2021). A subsequent study using an animal model showed that plate fixation resulted in a 35% reduction in torsional strength, indicating compromised long-term bone quality (Laftman et al., 1989). Consistent with these findings, as shown in Table 3, our study demonstrated that metal block augmentation increased the mean plate stress, especially in the type II and III configurations, although reducing proximal tibial bone stress, reinforcing a stress-shielding effect (Safavi et al., 2023). Because osteotomy-gap healing is predominantly cancellous, we contextualized our outputs by converting proximal tibial stress to equivalent microstrain using a cancellous elastic modulus (Hooke’s law, ε≈σ/E; E = 910 MPa, ν = 0.2) (Cowin and Doty, 2007). Across configurations, cancellous-equivalent microstrain decreased by approximately 5%–28% with block augmentation (e.g., uniplanar non-LHF 9.011 × 10^3^ → 7.143 × 10^3^; biplanar non-LHF 11.209 × 10^3^ → 8.132 × 10^3^, Supplementary Appendix S1), yet absolute values remained within the physiologic/osteogenic range (several thousand microstrain) rather than falling into a disuse zone (Sugiyama et al., 2012). This suggests that the osteogenic stimulus within the gap is likely preserved despite reduced mechanical input, aligning short-term mechanical benefit (enhanced stability, reduced interfragmentary motion) with maintenance of a biologically favorable strain environment. Given the substantially higher elastic modulus of cortical bone relative to cancellous tissue, cortical-equivalent strains beneath the plate could approach the lower hundreds of microstrains (i.e., near disuse levels). Clinically, the redistribution of load—plate stress increases while peri-implant bone stress decreases—may, if sustained, attenuate bone formation over time, a consideration particularly relevant in younger or high-demand patients. Reduced bone mass and structural integrity may pose challenges in future surgical procedures, including conversion to total knee arthroplasty, in which adequate metaphyseal bone stock is crucial for secure implant fixation (Yang et al., 2023). Because OWHTO hardware is typically retained for 1–2 years, we will consider a focused follow-up over this usual retention interval to evaluate cumulative stress shielding and any associated decrement in bone formation and to inform implant-removal timing in younger or high-demand patients.

This study has some limitations. First, all biomechanical analyses were based on a single FE model derived from the CT data of a female patient. Although this model provides high-resolution anatomical accuracy, it may not fully represent the anatomical variability observed across different patient populations, particularly in men or individuals with altered bone quality. Second, the loading protocol used in our study was limited to static compressive forces designed to simulate early postoperative weight-bearing conditions. However, actual OWHTO constructs are exposed to complex biomechanical environments involving cyclic, dynamic, and torsional loading. Such stresses play a critical role in assessing implant fatigue resistance and long-term mechanical performance (Bartolomeu et al., 2022; Weston et al., 2024). Future studies should incorporate these physiological loading conditions to enhance the translational validity and clinical relevance. Third, our simulation applied a fixed 60:40 medial-to-lateral load distribution. However, in clinical practice, the actual load distribution may vary depending on the degree of correction achieved through OWHTO. Thus, our findings may not fully reflect loading conditions across all correction angles. Fourth, microstrain values were estimated from mean proximal tibial stress for interpretive context, and we did not perform tissue-partitioned (cortical vs. cancellous) computations. As a result, local cortical under-loading beneath the plate may be underestimated, and site-specific effects should be interpreted with caution. Fifth, the surgical environment was idealized, and ligament, cartilage, and muscle forces were not modeled, lacking simulation of soft-tissue constraints or variations in osteotomy execution, such as hinge position or plate malposition. Finally, although metal block augmentation improved construct stability, the associated reduction in physiological loading may lead to stress shielding. The long-term clinical consequences, such as attenuation of bone remodeling or compromised metaphyseal bone stock relevant to future procedures (e.g., TKA) remain uncertain; we will consider follow-up over the typical hardware-retention interval (about 1–2 years after surgery) to evaluate cumulative stress shielding and any decrement in bone formation using radiographic or CT.

## 5 Conclusion

This study demonstrated that metal block augmentation enhanced biomechanical stability across all OWHTO configurations, with greater benefits observed in uniplanar osteotomies and in the presence of an LHF, where baseline stability was lower. The metal block uses a redistributed load—raising plate stress while lowering proximal tibia bone stress—consistent with stress shielding. Thus, although this option offers clear mechanical advantages in structurally vulnerable cases, its stress-shielding potential should be weighed in clinical decision-making, particularly for patients at risk of compromised bone quality or who require future surgical procedures.

## Data Availability

The raw data supporting the conclusions of this article will be made available by the corresponding author upon reasonable request and following approval by the institutional IRB.
