# Supplementary material for: Fixation stability and stress redistribution following metal block use in opening-wedge high tibial osteotomy: a finite element analysis
Source: Front Bioeng Biotechnol. 2025 Dec 17;13:1703140. doi: 10.3389/fbioe.2025.1703140 (PMC12754675; doi:10.3389/fbioe.2025.1703140)
Supplement: Supplementary file 1 [file Table1.docx]

Appendix 1. Equivalent microstrain in proximal tibia under tissue-modulus assumptions.

| Osteotomy type | Metal block | Cancellous-equivalent microstrain, ε (× 10^3)^ | | | | Cortical-equivalent microstrain, ε (× 10^3)^ | | | |
| --- | --- | --- | --- | --- | --- | --- | --- | --- | --- |
|  |  | Non-LHF | Type I LHF | Type II LHF | Type III LHF | Non-LHF | Type I LHF | Type II LHF | Type III LHF |
| Uniplanar | No | 9.011 | 8.681 | 4.396 | 7.033 | 0.482 | 0.465 | 0.235 | 0.376 |
| Uniplanar | Yes | 7.143 | 7.033 | 4.176 | 5.714 | 0.382 | 0.376 | 0.224 | 0.306 |
| Biplanar | No | 11.209 | 10.330 | 7.476 | 8.242 | 0.600 | 0.553 | 0.400 | 0.441 |
| Biplanar | Yes | 8.132 | 7.912 | 5.604 | 6.155 | 0.435 | 0.424 | 0.300 | 0.329 |

Hooke’s law. ε≈σ/E; cancellous E = 910 MPa. cortical E = 17.000 MPa; values rounded to nearest 10 με

Abbreviation: lateral hinge fracture; LHF, MPa; megapascal
